# Propensity matched analysis of minimally invasive versus conventional isolated aortic valve replacement

**DOI:** 10.1177/02676591211045802

**Published:** 2021-09-13

**Authors:** Shwe Oo, Amilah Khan, Jeremy Chan, Sanjay Juneja, Massimo Caputo, Gianni Angelini, Cha Rajakaruna, Hunaid A Vohra

**Affiliations:** Department of Cardiovascular Sciences, Bristol Heart Institute, University of Bristol, Bristol, UK

**Keywords:** aortic valve replacement, minimally invasive aortic valve replacement, MIAVR, mini-sternotomy, conventional aortic valve replacement, median sternotomy

## Abstract

**Objective:**

To analyse the early and mid-term outcome of patients undergoing conventional aortic valve replacement (AVR) versus minimally invasive via hemi-sternotomy aortic valve replacement (MIAVR).

**Methods:**

A single centre retrospective study involving 653 patients who underwent isolated aortic valve replacement (AVR) either via conventional AVR (*n* = 516) or MIAVR (*n* = 137) between August 2015 and March 2020. Using pre-operative characteristics, patients were propensity matched (PM) to produce 114 matched pairs. Assessment of peri-operative outcomes, early and mid-term survival and echocardiographic parameters was performed.

**Results:**

The mean age of the PM conventional AVR group was 71.5 (±8.9) years and the number of male (*n* = 57) and female (*n* = 57) patients were equal. PM MIAVR group mean age was 71.1 (±9.5) years, and 47% of patients were female (*n* = 54) and 53% male (*n* = 60). Median follow-up for PM conventional AVR and MIAVR patients was 3.4 years (minimum 0, maximum 4.8 years) and 3.4 years (minimum 0, maximum 4.8 years), respectively. Larger sized aortic valve prostheses were inserted in the MIAVR group (median 23, IQR = 4) versus conventional AVR group (median 21, IQR = 2; p = 0.02, SMD = 0.34). Cardiopulmonary bypass (CPB) time was longer with MIAVR (94.4 ± 19.5 minutes) compared to conventional AVR (83.1 ± 33.3; p = 0.0001, SMD = 0.41). Aortic cross-clamp (AoX) time was also longer in MIAVR (71.6 ± 16.5 minutes) compared to conventional AVR (65.0 ± 52.8; p = 0.0001, SMD = 0.17). There were no differences in the early post-operative complications and mortality between the two groups. Follow-up echocardiographic data showed significant difference in mean aortic valve gradients between conventional AVR and MIAVR groups (17.3 ± 8.2 mmHg vs 13.0 ± 5.1 mmHg, respectively; p = 0.01, SMD = −0.65). There was no significant difference between conventional AVR and MIAVR in mid-term survival at 3 years (88.6% vs 92.1%; log-rank test p = 0.31).

**Conclusion:**

Despite the longer CPB and AoX times in the MIAVR group, there was no significant difference in early complications, mortality and mid-term survival between MIAVR and conventional AVR.

## Introduction

The concept of minimally invasive aortic valve replacement (MIAVR) was first introduced by Rao and Kumar.^1^ They reported their experience in performing AVR via anterior right thoracotomy (ART).^1^ Since then, several minimally invasive techniques have been reported to be associated with improved early clinic outcomes.^2–4^ MIAVR has gained increasing popularity over the last 20 years by avoiding a full sternotomy and reducing surgical trauma.^5^ MIAVR approaches include right parasternal incision,^6^ trans-sternal incision,^7^ ART,^8^ J-shape or J Upper Hemi-sternotomy (UHS),^9^ and totally endoscopic AVR.^10^ Previous studies^11–18^ comparing conventional AVR and MIAVR with UHS has shown that MIAVR decreases blood loss and need for transfusion, provides better cosmesis, results in less post-operative pain, shorter ventilation time, less overall hospital stay and similar rates of short and long-term survival. However, the evidence for MIAVR is mostly based on observational clinical studies which are not well matched, constitute smaller numbers and lack adequate clinical and echocardiographic follow-up. In this study, we aim to undertake a propensity-matched (PM) analysis of the peri-operative results, mid-term survival and echocardiographic outcome of patients, who underwent conventional AVR versus MIAVR with UHS at our institution.

## Methods

This is a retrospective study on prospectively collected data including all adult patients (age >16 years) who underwent isolated AVR via a MIAVR with UHS or conventional AVR between August 2015 and March 2020. The exclusion criteria included, emergency AVR, MIAVR with ART, Ozaki procedure, redo or concomitant surgery. Preoperative characteristics, intraoperative and post-operative data such as short-term complications, follow-up echocardiographic data as well as mid-term survival were collected and analysed. Follow-up involved searching our online database system which stores statistics on the number of patients who are alive and that have died as well as date of death. Echocardiographic follow-up data was obtained using our Insignia online system which enables us to review imaging and accompanying reports. PM was performed using pre-operative characteristics to match the two groups. Pre- and post-matched patient characteristics and follow-up echocardiographic data were analysed using IBM SPSS Statistics for Windows, version 26 (IBM Corp., Armonk, N.Y., USA) and Microsoft excel. Institutional approval was obtained and patient consent was waived due to retrospective design.

## Surgical technique

All patients who underwent MIAVR had the UHS surgical technique performed. After general anaesthesia and establishment of sterile field, a UHS was performed into the right third or fourth intercostal space using the oscillating or reciprocating saw. Cardiopulmonary bypass (CPB) was then instituted between the ascending aorta and right atrium. After placement of aortic cross-clamp (AoX), antegrade blood cardioplegia was then given into the aortic root and then directly into right and left coronary artery ostia after aortotomy. Carbon dioxide was also used routinely. AVR was then performed under standard fashion with semi-continuous or interrupted pledgeted sutures.

## Statistical analysis

**Figure 1.**
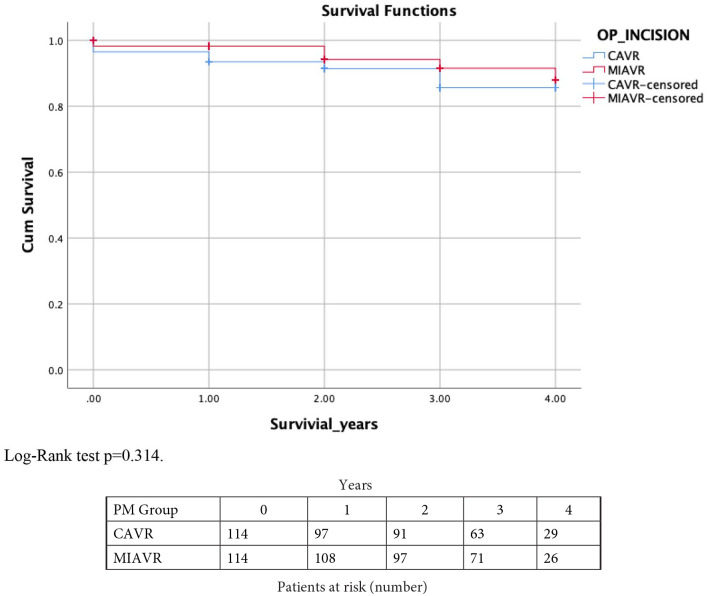
Kaplan Meier curve showing mid-term survival for the MIAVR and conventional AVR groups.

Using pre-operative characteristics, patients in the two groups were PM to decrease selection bias. Propensity score was calculated using logistic regression (LR). A total of 29 covariates were included in the LR model. Pre-operative characteristics which were inserted into propensity score matching included age, gender, body mass index, logistic euroscore, angina, previous myocardial infarction, previous percutaneous coronary intervention, diabetic, hypertension, smoking status, actual creatinine at time of surgery, dialysis for chronic renal failure, New York Heart Association classification score, chronic obstructive pulmonary disease/emphysema or asthma, neurological disease, neurological dysfunction, abnormal pre-operative heart rhythm, pre-op atrial fibrillation/flutter, ⩾1 coronary vessel disease, left main stem >50% diameter stenosis, severity of aortic valve stenosis (EOA in cm^2^, gradient mmHg), native aortic valve pathology, ejection fraction category, left ventricular ejection fraction, operative urgency, aortic valve haemodynamic pathology and first operator grade. Regarding pre-operative variables, including body mass index, previous myocardial infarction and hypertension had <1% missing data and pre-op atrial fibrillation had <2% data missing for both groups. Variables including >1 coronary vessel disease and left main stem disease had <4% data missing for the conventional AVR group. About 32% of data was missing for the severity of aortic valve stenosis (EOA in cm^2^) variable in the conventional AVR group and 16% in the MIAVR group. Similarly, 30% of the conventional AVR group had missing data for the severity of aortic valve stenosis gradient (mmHg) variable and 16% was missing for the respective MIAVR group. Overall though there was data missing it was not deemed significant enough to warrant inverse weighting of variables prior to analysis. Before matching, for pre-operative characteristics, all continuous data had the mean and standard deviation (SD), or median and interquartile range (IQR) calculated and proportions in percentages were also calculated for nominal data. Nearest neighbour PM method was used to match 114 MIAVR patients to 114 conventional AVR patients with the closest propensity score and these calculations were repeated (Table 1). Post-matching intra-operative and post-operative data and follow up echo data were also calculated including p value. For continuous data, the Shapiro-Wilk test was used to test for normality and p values were subsequently calculated using the Mann-Whitney *U* test for non-normally distributed variables. The standardised mean difference (SMD) was also calculated for continuous data. For nominal data, the Chi-squared test was used, as well as Fisher’s exact test. The Likelihood ratio was used where necessary and p < 0.05 were deemed significant. The mid-term survival of the patients was also reported with Kaplan-Meier survival analysis (Figure 1). Due to the small number of adverse outcomes in the two groups logistic regression was not carried out.

**Table 1.** Baseline patient characteristics of the two groups before and after propensity matching.

Variables	Unmatched groups						Matched groups			
	Group				p Value	Effect size (SMD)	Group		p Value	Effect size (SMD)
	(Conventional AVR) median sternotomy		(MIAVR) mini-sternotomy				(Conventional AVR) median sternotomy	(MIAVR) mini-sternotomy		
							*n* (114)	*n* (114)		
Pre-operative variables	Total *n*	Mean (±SD)/*n* (%)	Total *n*	Mean (±SD)/*n* (%)			Mean (±SD)/*n* (%)	Mean (±SD)/*n* (%)		
Age (years)	516	68.5 (±11.5)	137	71.3 (±9.4)	0.02	0.25	71.5 (±8.9)	71.1 (±9.5)	0.81	−0.04
Female	516	193 (37.4)	137	61 (44.5)	0.13		57 (50.0)	54 (47.4)	0.69	
BMI (kg/m^2^)	513	29.4 (±16.2)	136	28.2 (±5.4)	0.27	−0.08	27.9 (±5.2)	28.3 (±5.5)	0.54	0.07
Logistic Euroscore (%)	516	5.8 (±5.1)	137	5.7 (±3.7)	0.35	−0.02	5.6 (±4.1)	5.6 (±3.7)	0.78	0.00
Angina	516	210 (40.7)	137	66 (48.2)	0.12		55 (48.2)	56 (49.1)	0.90	
Previous MI	515	19 (3.7)	136	10 (7.4)	0.07		8 (7.0)	10 (8.8)	0.62	
Previous PCI	516	26 (5.0)	137	6 (4.4)	0.75		5 (4.4)	6 (5.3)	0.76	
Diabetic	516	94 (18.2)	137	22 (16.1)	0.56		21 (18.4)	22 (19.3)	0.87	
Hypertension	515	308 (59.8)	137	91 (66.4)	0.16		75 (65.8)	77 (67.5)	0.78	
Current smoker	516	37 (7.2)	137	7 (5.1)	0.39		4 (3.5)	4 (3.5)	1.00	
Actual creatinine at time of surgery (µmol/L)	516	89.0 (±40.2)	137	88.0 (±26.0)	0.84	−0.03	85.9 (±25.6)	87.1 (±26.6)	0.86	0.05
Dialysis for chronic renal failure*	516	2 (0.4)	137	0 (0.0)	1.00		0 (0.0)	0 (0.0)	NA	
NYHA 1–2	516	305 (59.1)	137	85 (62.0)	0.53		74 (64.9)	70 (61.4)	0.58	
NYHA 3–4		211 (40.9)		52 (38.0)			40 (35.1)	44 (38.6)		
COPD/emphysema or asthma	516	92 (17.8)	137	19 (13.9)	0.27		6 (5.3)	11 (9.6)	0.21	
Neurological disease*	516	35 (6.8)	137	12 (8.8)	0.43		8 (7.0)	8 (7.0)	1.00	
Neurological dysfunction	516	17 (3.3)	137	5 (3.6)	0.79		6 (5.3)	5 (4.4)	0.76	
Extracardiac arteriopathy	516	32 (6.2)	137	5 (3.6)	0.25		1 (0.9)	3 (2.6)	0.62	
Abnormal pre-operative heart rhythm*	516	67 (13.0)	137	16 (11.7)	0.68		8 (7.0)	12 (10.5)	0.35	
Pre-op atrial fibrillation/flutter	507	58 (11.4)	135	14 (10.4)	0.73		8 (7.0)	12 (10.5)	0.35	
⩾1 coronary vessel disease	500	29 (5.8)	137	8 (5.8)	0.99		7 (6.1)	8 (7.0)	0.79	
Left main stem >50% diameter stenosis	498	2 (0.4)	137	1 (0.7)	0.52		1 (0.9)	1 (0.9)	1.00	
Severity of aortic valve stenosis (EOA in cm^2^)	353	0.74 (±0.19)	115	0.74 (±0.19)	0.82	0.00	0.74 (±0.19)	0.74 (±0.19)	0.87	0.00
Severity of aortic valve stenosis (gradient mmHg)	359	73.3 (±24.0)	121	72.3 (±21.0)	0.85	−0.04	72.9 (±24.2)	72.0 (±21.3)	0.86	−0.04
Native aortic valve pathology										
Bicuspid	516	97 (18.8)	137	28 (20.4)	0.10		28 (24.6)	26 (22.8)	0.69	
Degenerative		393 (76.2)		106 (77.4)			85 (74.6)	86 (75.4)		
Infective endocarditis		14 (2.7)		0 (0.0)			0 (0.0)	0 (0.0)		
Rheumatic		1 (0.2)		1 (0.7)			0 (0.0)	1 (0.9)		
Other		11 (2.1)		2 (1.5)			1 (0.9)	1 (0.9)		
Ejection fraction category										
Good/fair	516	488 (94.6)	137	134 (97.8)	0.11		113 (99.1)	111 (97.4)	0.62	
Poor		28 (5.4)		3 (2.2)			1 (0.9)	3 (2.6)		
LVEF (%)	516	52.6 (±9.4)	137	54.4 (±6.6)	0.0001	0.20	55.6 (±8.2)	54.7 (±6.8)	0.39	−0.12
Operative urgency										
Elective	516	388 (75.2)	137	104 (75.9)	0.86		88 (77.2)	88 (77.2)	1.00	
Urgent		128 (24.8)		33 (24.1)			26 (22.8)	26 (22.8)		
Aortic valve haemodynamic pathology										
Stenosis	516	407 (78.9)	137	116 (84.7)	0.10		100 (87.7)	101 (88.6)	0.84	
Regurgitation		58 (11.2)		7 (5.1)			0 (0.0)	0 (0.0)		
Mixed		51 (9.9)		14 (10.2)			14 (12.3)	13 (11.4)		
First operator grade										
Consultant	516	305 (59.1)	137	101 (73.7)	0.002		97 (85.1)	86 (75.4)	0.07	

BMI: body mass index; LVEF: left ventricular ejection fraction; MI: myocardial infarction; NYHA: New York Heart Association classification; PCI: percutaneous coronary intervention.

Definitions: Dialysis for chronic renal failure: onset >6 weeks prior to cardiac surgery; Neurological disease: Transient ischaemic attack, Reversible ischemic neurological deficit, Stroke; Abnormal pre-operative heart rhythm: Atrial fibrillation, Atrial flutter, Complete heart block, Other abnormal heart rhythm.

## Results

A total number of 653 patients were included in this study, of which 137 (21.0%) and 516 (79%) had MIAVR and conventional AVR, respectively. PM resulted in 114 patients in each group for analysis. Pre-matched pre-operative data (Table 1) showed the mean age of patients undergoing MIAVR was significantly higher (71.3 ± 9.4 years vs 68.5 ± 11.5 years in conventional AVR; p = 0.02, SMD = 0.25). There was significant difference in the left ventricular ejection fraction (LVEF) between the two groups (52.6 ± 9.4% in conventional AVR vs 54.4 ± 6.6% in MIAVR; p = 0.0001, SMD = 0.20). After PM, the mean age was similar (71.5 ± 8.9 years and 71.1 ± 9.5 years for conventional AVR and MIAVR, respectively; p = 0.81, SMD = −0.04) and the mean LVEF of the conventional AVR group and MIAVR was 55.6 ± 8.2 years and 54.7 ± 6.8 years, respectively (p = 0.39, SMD = −0.12). The median aortic valve size (mm) used for conventional AVR and MIAVR was 21 (IQR = 2) and 23 (IQR = 4), respectively (p = 0.02, SMD = 0.34) (Table 2). Antegrade cardioplegia infusion was used in 67.5% of the conventional AVR patients and mixed (antegrade and retrograde) cardioplegia in 30.7% of the patients whereas all MIAVR patients received antegrade cardioplegia. Mean CPB time for MIAVR was significantly longer than conventional AVR (94.4 ± 19.5 minutes vs 83.1 ± 33.3 minutes respectively; p = 0.0001, SMD = 0.41). Moreover, AoX time was also longer in MIAVR (71.6 ± 16.5 minutes) compared to conventional AVR (65.0 ± 52.8; p = 0.0001, SMD = 0.17). Post-operative complications such as return to theatre for bleeding/tamponade (p = 0.50), deep sternal wound infection (p = 1.00), neurological dysfunction (p = 1.00), need for hemofiltration or dialysis (p = 1.00) showed no significant difference (Table 3). There were two in-hospital deaths in conventional AVR (1.8%) compared to none in MIAVR group (p = 0.50). The length of hospital stay was similar in both groups with a median of 6 days (IQR = 5) for the conventional AVR group and median of 7 days (IQR = 4) for the MIAVR group respectively; p = 0.81, SMD = −0.07). Echocardiographic follow-up data (Table 4) for the entire cohort showed that there was a significant difference in mean aortic gradients between conventional AVR and MIAVR (15.9 ± 7.6 mmHg vs 13.0 ± 5.0 mmHg, respectively; p = 0.004, SMD = −0.41). A similar trend in the mean aortic valve gradients was also seen in the matched population (conventional AVR 17.3 ± 8.2 mmHg vs MIAVR 13.0 ± 5.1; p = 0.01, SMD = −0.65). For all other variables including post-op LV function (pre-PM p = 0.07, post-PM p = 0.41), significant paravalvular leak (PVL), significant aortic regurgitation (AR) (p = 1.00), large pericardial effusions (pre-PM p = 1.00, post-PM p = 1.00) and number of reoperations (pre-PM p = 0.10, post-PM p = 0.64) were similar between the two groups pre and post matching. There was no significant difference between conventional AVR and MIAVR in the mid-term survival at 3 years (88.6% vs 92.1%; log-rank test p = 0.31), (Figure 1). Interestingly, 59.1% of conventional AVR were performed by consultants compared to 73.7% of MIAVR in pre-matched data (p = 0.002). However, after PM, the results showed 85.1% and 75.4% of conventional AVR and MIAVR, respectively, were performed by consultants (p = 0.07).

**Table 2.** Operative characteristics.

Variables	Matched groups					
	Group				p Value	Effect size (SMD)
	(Conventional AVR) median sternotomy		(MIAVR) mini-sternotomy			
Intra-operative variables	Total *n*	Mean (±SD)/*n* (%)	Total *n*	Mean (±SD)/*n* (%)		
Aortic valve implant type						
Mechanical	114	9 (7.9)	114	12 (10.5)	0.49	
Biological		105 (92.1)		102 (89.5)		
Aortic valve/ring size (mm)						
Median (IQR)	114	21 (2)	114	23 (4)	0.02	0.34
Cardioplegia temperature						
Cold	114	114 (100.0)	114	113 (99.1)	1.00	
Warm		0 (0.0)		1 (0.9)		
Cardioplegia infusion mode						
Antegrade	114	77 (67.5)	114	114 (100.0)	0.0001	
Retrograde		2 (1.8)		0 (0.0)		
Mixed (Antegrade;Retrograde)		35 (30.7)		0 (0.0)		
CPB time (minutes)	111	83.1 (±33.3)	110	94.4 (±19.5)	0.0001	0.41
CPB time excluding sutureless (minutes)	108	83.4 (±33.7)	93	97.2 (±19.2)	0.0001	0.49
AoX time (minutes)	111	65.0 (±52.8)	108	71.6 (±16.5)	0.0001	0.17
AoX time excluding sutureless (minutes)	108	65.5 (±53.4)	92	74.5 (±15.8)	0.0001	0.22
Sutureless valves						
PCV	114	3 (2.63)	114	12 (10.53)	0.001	
INTY		0 (0.00)		5 (4.39)		

AoX: aortic cross-clamp; CPB: cardiopulmonary bypass; INTY: intuity; PCV: perceval.

**Table 3.** Post-operative results.

Variables	Matched groups					
	Group				p Value	Effect size (SMD)
	(Conventional AVR) median sternotomy		(MIAVR) mini-sternotomy			
Post-operative variables	Total *n*	Mean (±SD)/*n* (%)	Total *n*	Mean (±SD)/*n* (%)		
Return to theatre*	114	6 (5.3)	114	3 (2.6)	0.50	
Deep sternal wound infection	114	1 (0.9)	114	0 (0.0)	1.00	
New post-op neurological dysfunction*	114	0 (0.0)	114	1 (0.09)	1.00	
New post-op hemofiltration or dialysis	114	1 (0.9)	114	0 (0.0)	1.00	
Patient deceased at discharge	114	2 (1.8)	114	0 (0.0)	0.50	
Length of hospital stay (days)	114	6 (5)	114	7 (4)	0.81	−0.07
Median (IQR)						

Definitions: new post-op neurological dysfunction: Transient Stroke, permanent stroke; return to theatre: for bleeding, tamponade or other cardiac problem.

**Table 4.** Echocardiographic outcomes at latest follow-up.

Variables	Unmatched groups						Matched groups					
	Group				p Value	Effect size (SMD)	Group				p Value	Effect size (SMD)
	(Conventional AVR) median sternotomy		(MIAVR) mini-sternotomy				(Conventional AVR) Median sternotomy		(MIAVR) mini-sternotomy			
Follow-up echo variables	Total *n*	Mean (±SD)/*n* (%)	Total *n*	Mean (±SD)/*n* (%)			Total *n*	Mean (±SD)/*n* (%)	Total *n*	Mean (±SD)/*n* (%)		
Poor LV function	269	10 (3.7)	89	0 (0.0)	0.07		54	1 (1.9)	77	0 (0.0)	0.41	
Mean aortic gradient (mmHg)	219	15.9 (±7.6)	71	13.0 (±5.0)	0.004	−0.41	45	17.3 (±8.2)	62	13.0 (±5.1)	0.01	−0.65
Significant PVL	269	0 (0.0)	89	0 (0.0)	N/A		54	0 (0.0)	77	0 (0.0)	N/A	
Significant AR	269	1 (0.4)	89	0 (0.0)	1.00		54	0 (0.0)	77	0 (0.0)	N/A	
Large (>20 mm) pericardial effusion	269	6 (2.2)	88	2 (2.3)	1.00		54	1 (1.9)	76	1 (1.3)	1.00	
Reoperation	269	2 (0.7)	89	3 (3.4)	0.10		54	1 (1.9)	77	3 (3.9)	0.64	

AR: aortic regurgitation; LV: left ventricular; PVL: paravalvular leak.

## Discussion

Our findings of longer CPB and AoX times with MIAVR compared to conventional AVR are in line with similar results in the literature.^3,19^ Despite this, early post-operative complications and survival were comparable between the two groups. There is no doubt that any kind of minimally invasive cardiac surgery is more challenging when compared with conventional procedure via median sternotomy. This is mainly due to a limited incision and reduced operative space that lead to restricted manoeuvrability.^20^ Given that increased CPB and AoX times are known to be associated with adverse early outcomes, our study with MIAVR did not support this notion. Whether this leads to enhanced systemic inflammatory response or degree of myocardial damage at molecular level remains to be explored and more work is required in this area. Despite the complexity of MIAVR operations and risk of subsequent complications, MIAVR did not increase early mortality compared to conventional AVR, reasons may be due to technical maturation of the procedure.^21^ Though previous studies found reduced length of hospital stay and post-operative complications with MIAVR, our post-operative findings were similar for both groups indicating similar safety for both operations.^22^ Some authorities also believe that higher mid-term mortality during follow-up may occur due to increased AoX time leading to inadequate cardio-protection and reduced LV function. We did not demonstrate this but larger studies with longer follow-up may be required to further determine this.

A surprising finding is that larger valve prostheses were inserted in the MIAVR group with significantly lower mean aortic valve gradients compared to conventional AVR. The reason for this is not completely understood but the higher insertion of rapid deployment valves (RDV) in our MIAVR series compared with conventional AVR may have contributed to this.^23,24^

A steep learning curve for MIAVR has been reported by several authors, even amongst the most experienced surgeons.^14^ Hence, it is unsurprising to see in the literature that mostly MIAVR are performed by consultants. However, it has been reported that a MIAVR training programme can be established without compromising patient safety.^25^ Masuda et al.^26^ suggested approximate 40–50 cases were required to overcome the learning curve which may not be the ideal stage to train residents. Similar data were also reported by Murzi et al.^27^ In our institution, nearly three quarters of the MIAVRs were performed by consultant surgeons. However, an increasing trend was seen in trainees performing MIAVRs in our unit with comparable results in pre-PM data.

One of the limiting factors of our study is that it is based on single centre retrospective data, analysing the results of only one type of MIAVR. Therefore, propensity matching was conducted to decrease selection bias. Furthermore, as a regional cardiac surgical centre, some patients had their follow-up echocardiography done in the referral centres of which not all follow up data was accessible. Therefore, the echocardiographic analysis was performed with a smaller group of patients.

## Conclusion

Our results have shown that larger aortic valve prostheses are being implanted in the MIAVR group with lower mean aortic valve gradients. The complication rate, early and mid-term mortality were comparable to conventional AVR despite longer CPB and AoX times with MIAVR. Follow-up echocardiographic data currently shows no difference; however, analysis of long-term follow-up data with larger number of matched patients is required to ascertain late outcomes.
